# Xylem anatomical responses of *Larix Gmelinii* and *Pinus Sylvestris* influenced by the climate of Daxing’an mountains in Northeastern China

**DOI:** 10.3389/fpls.2023.1095888

**Published:** 2023-01-30

**Authors:** Taimoor Hassan Farooq, Sumaira Yasmeen, Awais Shakoor, Muhammad Farrakh Nawaz, Muhammad Haroon U. Rashid, Sarir Ahmad, Majeeda Rasheed, He Li, Qian Li

**Affiliations:** ^1^ Bangor College China, a Joint Unit of Bangor University and Central South University of Forestry and Technology, Changsha, Hunan, China; ^2^ Center for Ecological Research, Northeast Forestry University, Harbin, China; ^3^ Teagasc, Environment, Soils and Land Use Department, Johnstown Castle, Co, Wexford, Ireland; ^4^ Department of Forestry and Range Management, University of Agriculture, Faisalabad, Pakistan; ^5^ Department of Forestry, University of Agriculture, Dera Ismail Khan, Pakistan; ^6^ Department of Life Sciences, Khwaja Fareed University of Engineering and Information Technology, Rahim Yar Khan, Pakistan; ^7^ College of Forestry, Central South University of Forestry and Technology, Changsha, Hunan, China

**Keywords:** cell wall thickness, hydraulic properties, latitudinal gradient, ring width, tracheid size, wood anatomical characteristics

## Abstract

Wood anatomy and plant hydraulics play a significant role in understanding species-specific responses and their ability to manage rapid environmental changes. This study used the dendro-anatomical approach to assess the anatomical characteristics and their relation to local climate variability in the boreal coniferous tree species *Larix gmelinii* (Dahurian larch) and *Pinus sylvestris* var. *mongolica* (Scots pine) at an altitude range of 660* m* to 842 m. We measured the xylem anatomical traits (lumen area (LA), cell wall thickness (CWt), cell counts per ring (CN), ring width (RW), and cell sizes in rings) of both species at four different sites Mangui (MG), Wuerqihan (WEQH), Moredagha (MEDG) and Alihe (ALH) and investigated their relationship with temperature and precipitation of those sites along a latitude gradient. Results showed that all chronologies have strong summer temperature correlations. LA extremes were mostly associated with climatic variation than CWt and RWt. MEDG site species showed an inverse correlation in different growing seasons. The correlation coefficient with temperature indicated significant variations in the May-September months at MG, WEQH, and ALH sites. These results suggest that climatic seasonality changes in the selected sites positively affect hydraulic efficiency (increase in the diameter of the earlywood cells) and the width of the latewood produced in *P. sylvestris*. In contrast, *L. gmelinii* showed the opposite response to warm temperatures. It is concluded that xylem anatomical responses of *L. gmelinii* and *P. sylvestris* showed varied responses to different climatic factors at different sites. These differences between the two species responses to climate are due to the change of site condition on a large spatial and temporal scale.

## 1 Introduction

Environmental changes are likely to affect critical ecological processes that affect key natural resources, especially in northern ecosystems (Baltzer et al., 2014; Carrer et al., 2017). Daxing’anling mountains of Inner Mongolia are major climate change hot spots that belong to the sub-frigid zone with a pronounced cold temperate and continental monsoon climate (Huang et al., 2020; Zheng et al., 2022). Moroever, the Mongolian forests and grasslands of northern regions have also been degraded by dry wind, desertification, soil salinization, and erosion. Environmental changes drastically impact this fragile area and its vicinity, especially in the context of global warming and seasonal water deficit (Bao, 2015; Carvalho et al, 2022). Northeastern region’s forests are affected by reduced rainfall, increased temperatures, and frequent periods of extreme dryness; therefore, studies of forests in northeast China are critical because of their sensitivity and vulnerability to harsh conditions in the frozen ground in the context of regional and global climate change (Zhang et al., 2018).

The key advantage of anatomical features’ are related to tree physiology and their strong connection to hydraulic properties (Souto-Herrera et al., 2018). Wood anatomy and plant hydraulics play a significant role in understanding species-specific responses and the ability to manage rapid environmental changes (Balzano et al., 2020). At the start of the season, earlywood vessels are most efficient for water transfer. At the same time, latewood primarily consists of much smaller vessels developed under the more constrained water conditions of late spring and summer (Aloni, 2015). In coniferous species, most wood components are tracheid, providing both vascular and mechanical support functions (Emanuele et al., 2014). Trees can adapt to changes in external conditions with a high degree of plasticity and variability in newly formed cells. As a result, these changes are recorded in the anatomical characteristics of tree rings (Fonti et al., 2013).

Previous studies have shown that tree ring width depends on changes in climatic conditions and the geographic location of the site (Creasman, 2011). Trees are predicted to increase their elevations; as the environment warms, some changes in treeline elevation have been observed and attributed to global warming (García-Cervigón et al., 2020). In conifers, seasonal environmental conditions hugely determine the ring width and tracheid size; these anatomical parameters affect the intra-annual dynamics of radial growth (Rahman et al., 2016). Mature tracheid is formed at different time intervals, which causes differences in cell size, cell wall thickness, and cell number. Therefore, they are good indicators of intra-annual growth rate and tree-ring structure changes (Fonti et al., 2013; Benkova et al., 2015).

Recently, based on the internal annual characteristics of the tree rings, there has been a growing interest in deciphering the effect of the climate on a whole xylogenetic process, e.g., the sequence of phases that contribute to the environment, through photosynthetic and cambial activities, leads to the creation of new wood tissues ( Rossi et al., 2012; Cuny et al., 2014; 2015). Recent advances in wood anatomy have significantly improved the accuracy and efficiency of xylem structural measurements (Von Arx et al., 2016; Prendin et al., 2017). However, the meristematic processes and mechanisms of radial growth influenced by environmental changes are still poorly understood (Körner, 2015). Tree-ring anatomy is a promising method for uncovering the process of wood formation and exploring the characteristics of cell anatomy. This study analyzed the (1) xylem plasticity of two widespread evergreen tree species *Larix gmelinii* (Dahurian larch) and *Pinus sylvestris* var. *mongolica* (Scots pine), in the Daxing’anling mountains to climatic variability and (2) determine the intra-annual variation of xylem responses to climatic parameters. Xylem anatomical traits such as (lumen area (LA), cell wall thickness (CWt), cell counts per ring (CN), ring width (RW), and cell sizes in rings) were measured of both species at four different sites Mangui (MG), Wuerqihan (WEQH), Moredagha (MEDG) and Alihe (ALH) to investigate their relationship with temperature and precipitation of those sites along a latitude gradient.

## 2 Materials and methods

### 2.1 Study area

**Figure 1:**
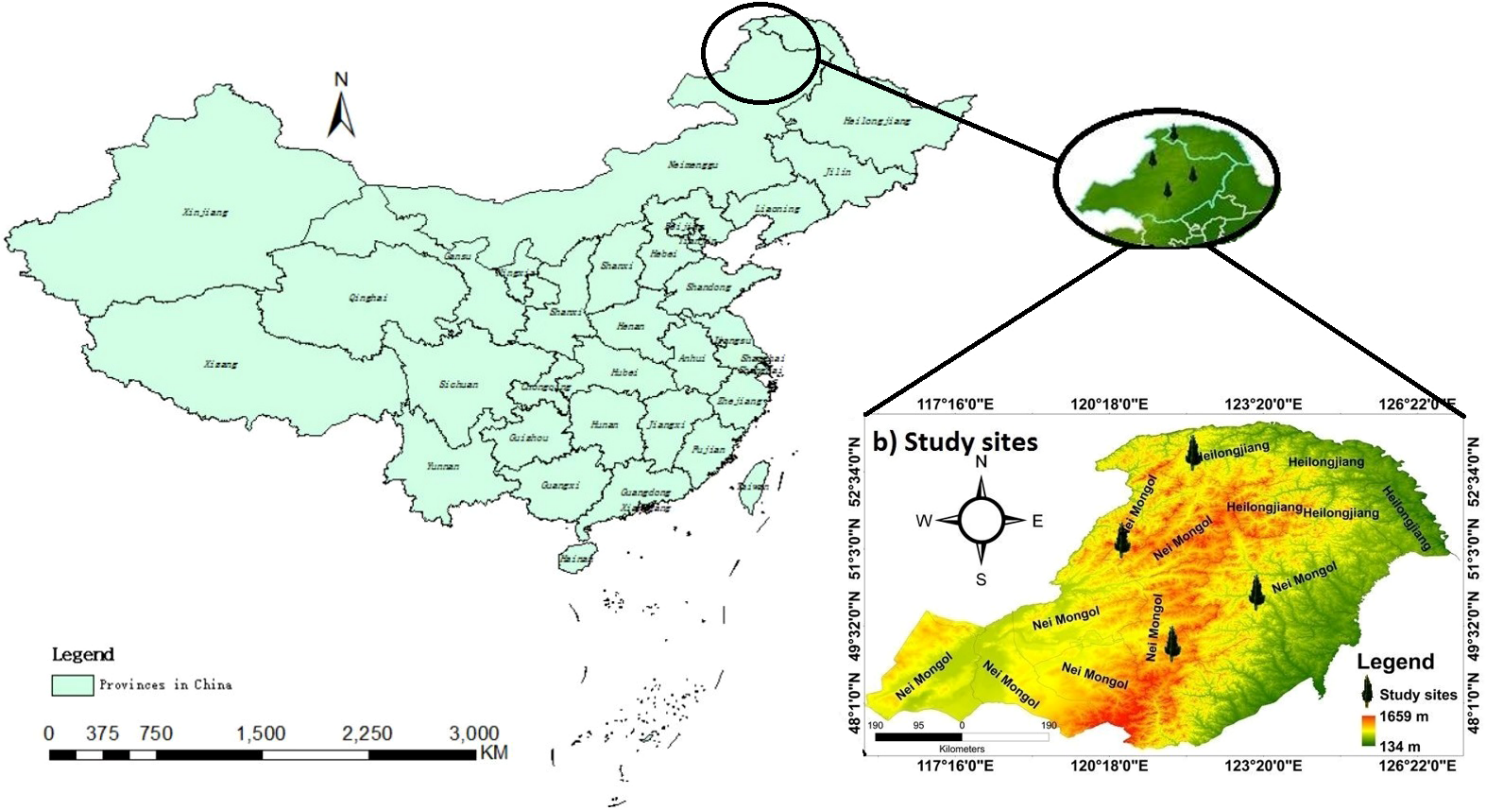
Location map of the four sampling sites in northeast China.

**Figure 2:**
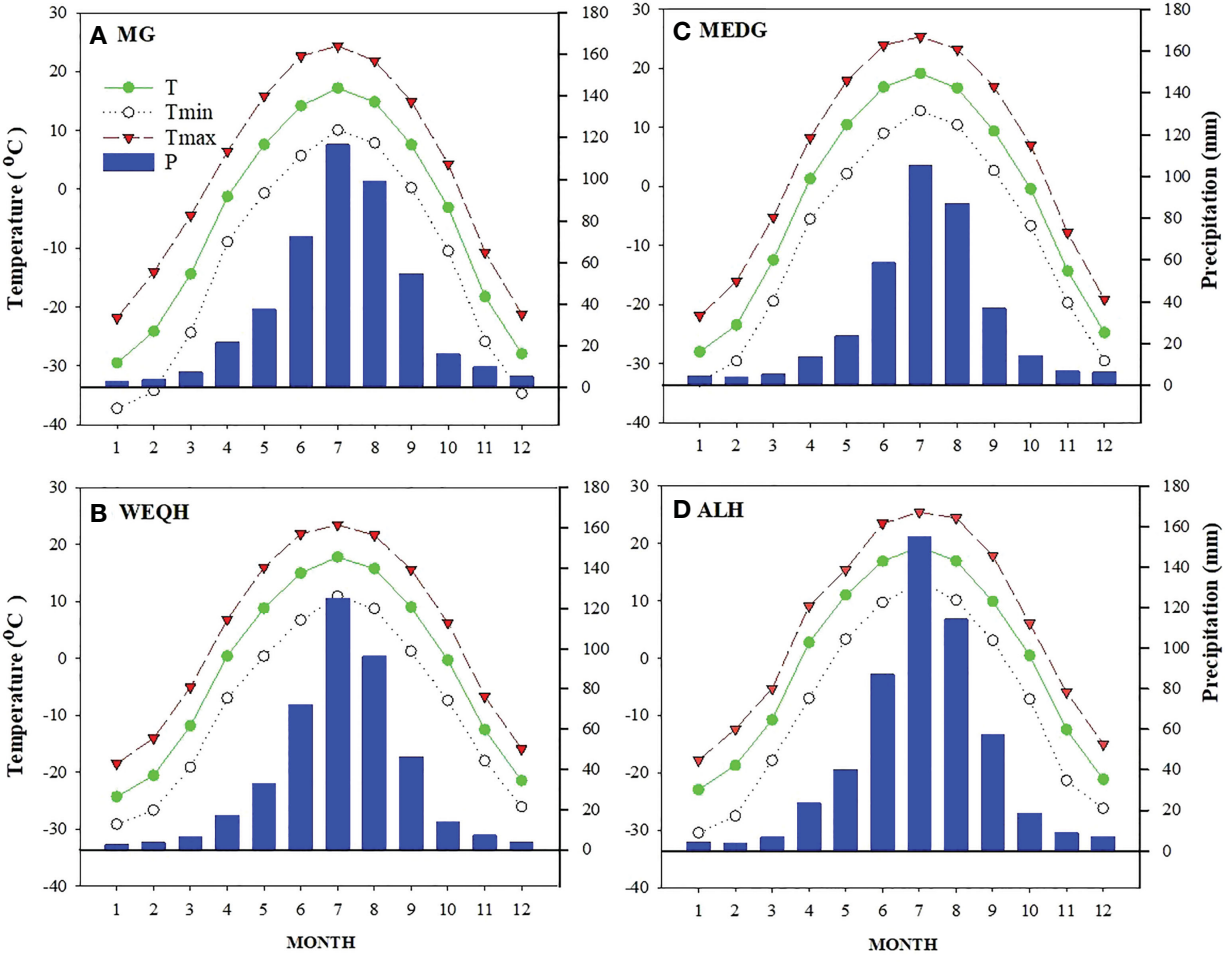
Weather profile of four sampling sites (MG, WEQH, MEDG, ALH) located at the northern Daxing’anling mountains, China. The period of each station used to calculate the monthly mean value was the same as in **Table 1**.

The Daxing’an Mountains are located in northeast China, extending about 1200 km from north to south. The average width is around 200 km, and the elevation is about 573 m. Its geographical range is between 49.12°N and 52.88°N (**Figure 1**). The climate of the study area is semi-humid and continental monsoon (Tian et al., 2017). This area has been covered with ice and snow for about half a year. Mean annual temperatures range from -0.8°C to -5.5°C. Annual precipitation varies between 437- 460 mm, with about 80% occurring from June to September (**Figure 2**). The same study area was also used for our published article (Yasmeen et al., 2019).

The study area belongs to the southern margin of the boreal forest. The primary forests are deciduous-coniferous forests having *L. gmelinii*, *P. sylvestris* var. *mongolica*, and other common tree species, including *Quercus mongolica, Betula platyphylla*, *Calamagrostic turczaninowii*, *Rosa davurica*, *Pinus pumila*, *Salix matsudan* and *Populus davidiana* (Gao et al., 2019). Climate data (monthly mean temperature and total precipitation) data set was obtained from the KNMI climate explorer (CRU grid TS 4.01) (http://climexp.knmi.nl). Climatic data and other relevant information about sampling sites are shown in (**Table 1**) (Yasmeen et al., 2019).

**Table 1:** Information of sampling sites at the Daxing’anling Mountains, northeast China.

	Sites	Elevation (m)	Latitude (°E)	Longitude (°N)	MLa (µm^2^)	MRW (mm)	MCN	MAT (°)	TAP (mm)
*Larix gmelinii*	MG	699.9	52.88	121.95	33.8 ± 2.1	0.095	60.8	-3.8	443.9
	WEQH	747.0	49.12	121.54	39.5 ± 4.4	0.115	62.2	-1.6	423.3
	MEDG	842.7	51.16	120.55	42.2 ± 9.4	0.046	30.7	-1.6	362.9
	ALH	660.0	50.13	123.20	35.2 ± 6.7	0.228	160.0	-0.6	524.6
*Pinus sylvestris* var. *mongolica*	MG	699.9	52.88	121.95	39.5 ± 8.3	0.096	62.1	-3.8	443.9
	WEQH	747.0	49.12	121.54	41.4 ± 3.5	0.458	85.0	-1.6	423.3
	MEDG	842.7	51.16	120.55	44.1 ± 7.1	0.057	7.9	-2.5	362.9
	ALH	660.0	50.13	123.20	48.0 ± 4.4	0.129	87.5	-0.6	524.6

MLa, mean lumen area, MRW, mean ring width, MCN, mean cell number, MAT, mean annual temperature of 2016, TAP, total annual precipitation of 2016.

### 2.2 Plant material and sampling

This work was carried out in the central and northern parts of Daxing’an Mountain. *P. sylvestris* and *L. gmelinii* species were taken as study material. The targeted species were distributed from 660-842 m above sea level. Two 20 × 20 m experimental plots were delineated at each sampling site for both species. Height and diameter at breast height (DBH) were taken for all the trees in each stand, and five standard trees were selected for tree core sampling from each stand. Tree cores were extracted at breast height (1.3 m) with a Pressler borer close to the local latitudinal limit (i.e., between 660-843 m). From each sampling site, 25 samples were taken (5 from each tree).

### 2.3 Anatomical analysis

A transmitted light microscope (Olympus-IX83, Olympus Co., Ltd., Beijing, China) was used for detailed anatomical studies. Xylem cells were differentiated into different cell types by observing cell division, cell differentiation, and programmed death of a cell. Microcores were split into 4-5 cm long, according to the longitudinal extent of cells. To prepare microcores, hand sliding rotary microtome (KD-2258, Ningbo Hionotek Instrument Co. Ltd., Ningbo, China) was used to obtain 10-20 µm thick sections. It was crucial for cutting microsections to facilitate high-quality sections for image analysis (Gärtner et al., 2015). Depending on the specific aim, sections were cut transverse. Different cell dimensions of the microsections were measured by taking photographs under the microscope using free image analysis software. The following wood-anatomical traits were measured in the transversal xylem sections; mean lumen area (LA) and radial cell wall thickness (CWt). These factors were chosen because of their higher year-to-year variability than the tangential dimensions (Vysotskaya and Vaganov, 1989). The time interval of sample collection was about two weeks.

### 2.4 Protocol for the preparation of high-quality slides

For sample collection, we used a trephor with an opening of 2 mm depending on the thickening of the bark of the tree. After removing microcore sections from trees, samples were immediately preserved in an Eppendorf tube filled with the preserving solution of formalin acetic acid-ethanol (500 mL) (Tardif and Conciatori, 2015; Von Arx et al., 2016). For making clear and high-quality images of micro core slides, we dipped micro cores in a series of ethanol solutions, anhydrous ethanol, and dimethyl benzene for 4-days. For that purpose, the tissues were dipped in 30, 50, 70, and 85% ethanol solutions for 90-120 min, while 95% ethanol solution was used for 60-90 min. Anhydrous ethanol solution was used for 60-90 minutes. After that, the dimethylbenzene and ethanol were used at a ratio of 1:1 for an hour. In the end, paraffin blocks were made for cutting the micro-core sections. Softening solutions and stains were also made for slide preparation. It was put into a softening solution (glycerine:70% ethanol) to soften micro-core wood. For good-quality image analysis of xylem cell structures, we selected the Leica^®^ Disposable Microtome Blades 819, and gelatin solution was used (gelatin, glycerol, and water) to fix cores properly. We cut the transversal micro core slices between 10-20 µm thick using Leica rotary microtome (KEDEE, Leica Biosystem, Buffalo Grove, Illinois USA).

### 2.5 Paraffin embedding for tissues of micro cores with safranin

Histological paraffin techniques were used for embedding, and the samples were put in the melted condition of paraffin wax. Micro cores were dipped in dimethyl benzene solution 0.88 g/mL for 12 min. A series of solutions were used i.e., anhydrous ethanol: dimethyl benzene (1:1), anhydrous ethanol, 95% ethanol, 85% ethanol, 75% ethanol for 6 min, subsequently. Afterward, the tissues were dipped in 1% safranin solution for 4 h, followed by 60 s in water, and then used a series of 30%, 50%, 70%, and 80% anhydrous ethanol solutions for 60 s. In the last two steps, anhydrous ethanol and benzene were used at the ratio (of 1:1) for 60 s. After this, the tissues were kept on the slides, and Canada balsam (CAS: 8007-47-4, Sigma-Aldrich, Darmstadt, Germany) was used for preservation and covered with a coverslip for image analysis. Images were captured by an Olympus DP-73 microscope (Olympus U-TV0.5XC-3) SN 4A01028 mounted on a computer. Then measurements of high-quality images were taken by IPWIN-32 (Image-Pro plus). Each micro core included several xylem layers (4-5 in fast-growing and 37-45 in slow-growing seasons). The average number of tree rings in all species was 11, so 2006-2016 was selected for comparison purposes. Moreover, tree rings were visually cross-dated along 5 lines in each annual ring. Anatomical parameters included ring width (RW), early wood width (EW), latewood width (LW), lumen area (LA), and cell wall thickness (CWt). Earlywood and latewood were measured by taking an average of 5 lines of tracheid diameter classified as earlywood and latewood. Intraspecific features were measured by comparing trees of the same species at different elevations and interspecific comparisons of different species at the same or different elevations. Anatomical parameters were calculated separately for earlywood and latewood e.g., lumen area of early wood tracheid (la EW) and latewood tracheid (la LW).

### 2.6 Statistics and climate growth relationship

A two-way analysis of variance (ANOVA) was performed to determine the differences in xylem anatomical features among four sites and species. Results showed significant differences, so Tukey’s pairwise comparison was used by LSD to determine the significance. The relationship between anatomical traits and tree rings was tested by Pearson correlation by using Past software. climatic influence on xylem anatomical features was measured using CRU TS 4.0 monthly climate data of mean temperature and precipitation. The climate growth associations with anatomical parameters were also quantified by Pearson correlation.

## 3 Results

Significant statistical differences (*P* < 0.05) were observed when La-EW and La-LW were analyzed among all the sites and species treatments. Overall, for both species, the highest La-EW and La-LW were observed at the ALH site, followed by the MG site. For CWt-EW, the highest and lowest values were observed at the MG site for *L. gmelinii* (1.81 ± 0.26 µm) and *P. sylvestris* (1.17 ± 0.31 µm), *respectively*. No significant difference was observed for CWt-EW values of *P. sylvestris* among WEQH, MEDG, and ALH sites. The same pattern of CWt-EW was observed for CWt-LW; however, significant differences were observed among sites for both species (**Table 2**). Regarding RW-EW and RW-LW, for *L. gmelinii*, the highest value was observed at the ALH site and the lowest at MEDG. Whereas for *P. sylvestris*, the highest value was observed at the WEQH and the lowest at MEDG (**Table 2**).

**Table 2:** Wood and tracheid properties of *Larix gmelinii* and *Pinus Sylvestris* L.

Species	Sites	La-EW (µm^2^)	La-LW (µm^2^)	CWt-EW (µm)	CWt-LW (µm)	RW-EW (10^-2^ mm)	RW-LW (10^-2^ mm)
*Larix gmelinii*	MG	211.7 ± 42.2 b	109.3 ± 30.5 b	1.82 ± 0.26 a	2.67 ± 0.39 a	1.36 ± 0.28 ab	0.56 ± 0.19 ab
	WEQH	157.8 ± 13.0 d	72.6 ± 19.2 bc	1.78 ± 0.36 bc	2.41 ± 0.41 bc	1.52 ± 0.50 b	0.88 ± 0.34 b
	MEDG	191.6 ± 30.1 c	62.6 ± 16.4 c	1.68 ± 0.38 c	2.47 ± 0.51 c	0.56 ± 0.20 ab	0.21 ± 0.14 bc
	ALH	310.6 ± 28.9a	118.0 ± 17.7 a	1.81 ± 0.13 a	2.54 ± 0.24 b	2.61 ± 0.76 a	1.74 ± 0.43 a
*Pinus sylvestris* L. var. *mongolica*	MG	229.9 ± 32.9 b	122.9 ± 16.7 a	1.17 ± 0.31 b	1.75 ± 0.32 bc	1.96 ± 0.63 b	0.59 ± 0.37 b
	WEQH	140.1± 16.8 d	56.1 ± 16.5 b	1.38 ± 0.31a	1.97 ± 0.19 a	3.37 ± 0.35a	1.51 ± 0.24 a
	MEDG	170.1 ± 17.4 c	56.8 ± 13.2 b	1.38 ± 0.31 a	1.84 ± 0.44 b	0.60 ± 0.18c	0.27 ± 0.25 ac
	ALH	309.3± 24.4 a	124.6 ± 18.1 a	1.38 ± 0.31 a	1.68 ± 0.19 c	1.50± 0.27ab	0.60 ± 0.21 c

Values are means ± standard deviations. Different letters within columns indicate significance at p ≤ 0.05 according to Fisher’s least significant difference (LSD) test. La-EW, Lumen area of earlywood, La-LW, Lumen area of latewood, CWt-EW, Cell wall thickness of earlywood, CWt-LW, Cell wall thickness of latewood, RW-EW, Ring width of earlywood, RW-LW, Ring width of latewood.

**Figure 3:**
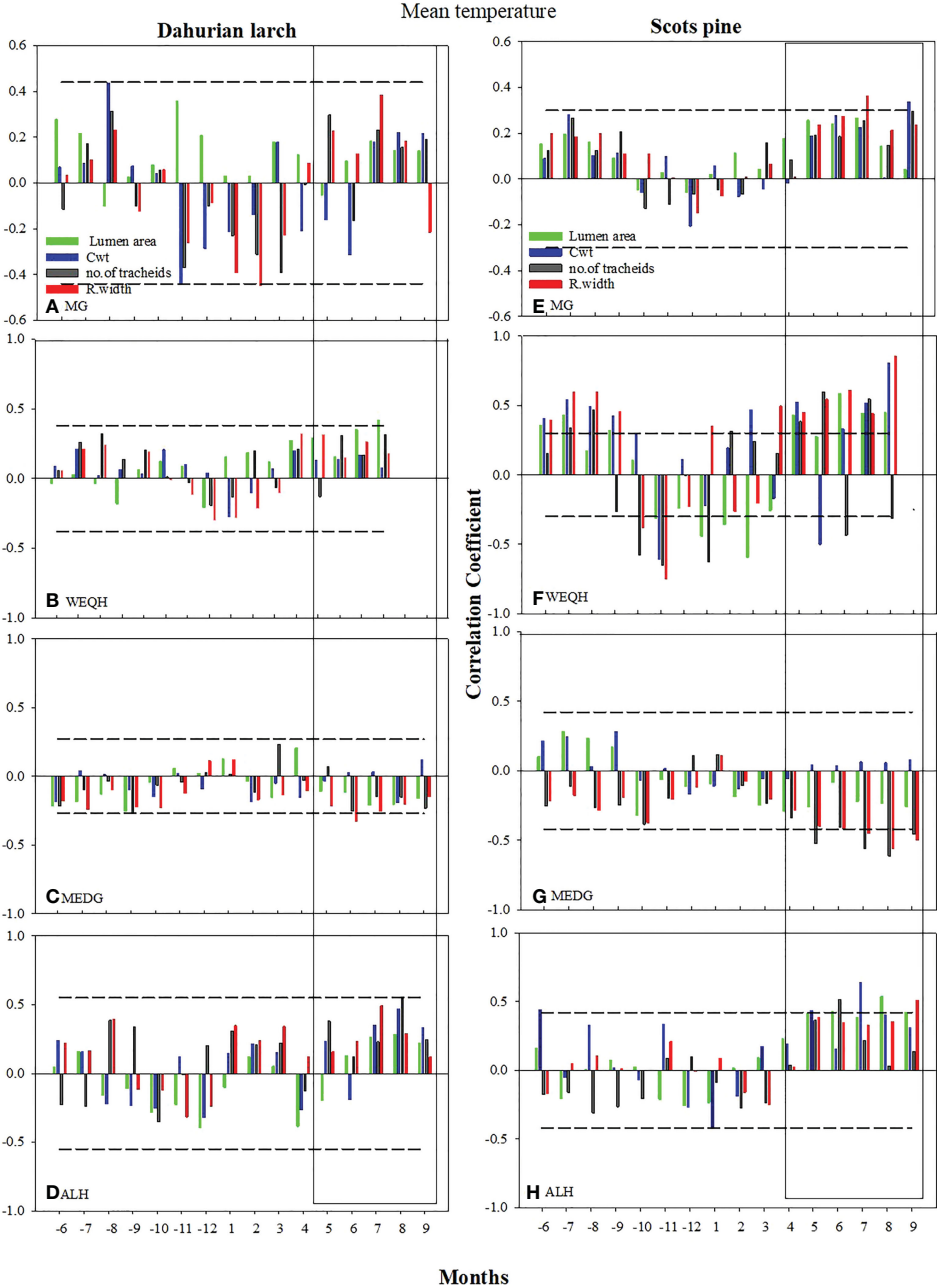
Climate growth relationship between the chronologies of xylem anatomical traits and monthly mean temperature (Grid Cru TS.4 for the period 2006-2016) from previous May – to current September. Horizontal dotted line shows most significant level (*p ≤ 0.05*). The rectangles indicate the period with mostly strong correlations.

The correlation of xylem anatomical parameters with temperature indicated significant variations (*P* < 0.05) for both species in May-September at MG, WEQH, and ALH sites, except MEDG. The correlation of MLa, RW, and CWt with mean temperature reached a higher level at the MG site in *P. sylvestris* in June, August, and September and MLa, CWt, and RWt showed a significant increase in the current growing season. No significant growth was observed for *L. gmelinii* at the MG site in June, August, and September; however, at the WEQH and ALH sites, *P. sylvestris* showed a significant increase in growth rate in these months. At MEDG site, both species showed an inverse relationship with temperature in the growing season (**Figure 3**). MLa and ring width showed a stronger response than the number of tracheids and cell wall thickness. Cell anatomical traits appeared in a stronger relationship with the temperature at WEQH and ALH. Despite the typical response from May to September, *L. gmelinii* was slightly more affected by temperature (**Figure 3**).

**Figure 4:**
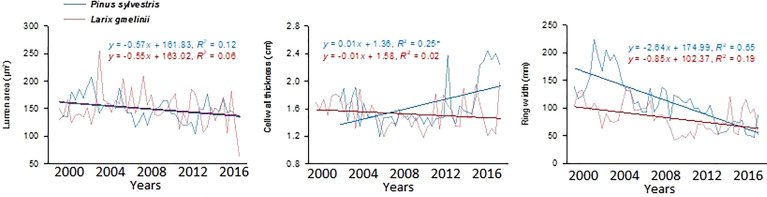
Mean lumen size, cell wall thickness, and tree ring width chronologies of *Pinus sylvestris* (Scots pine) and *Larix gmelinii* (Dahurian Larch) between the period 2000-2016.

**Figure 5:**
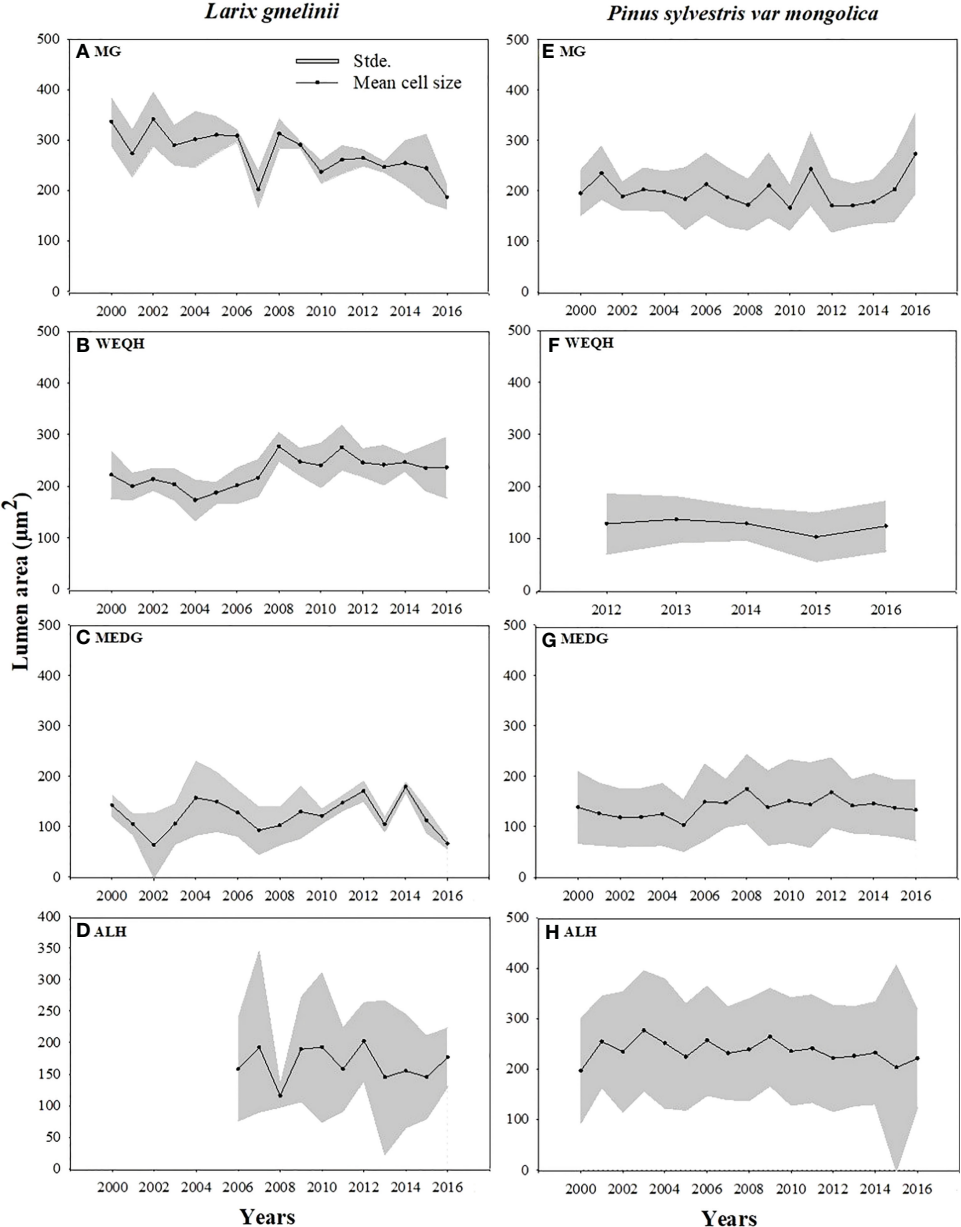
Mean standardized trachediogram of *Pinus sylvestris* (Scots pine) and *Larix gmelinii* (Dahurian Larch) during 2000-2016 except for WEQH (*P. sylvestris*) and ALH (*Larix gmelinii*) which have 2012-2016 and 2006-2016 data, respectively.

Growth trends were made by comparing MLa, CWt, and tree RW trends of the last five decades. MLa showed a linear trend for both species. However, it was statistically non-significant for *P. sylvestris* (R^2^ = 0.12) (**Figure 4**). The CWt of *P. sylvestris* showed an increasing trend across the years, whereas *L. gmelinii* showed a significant decreasing trend in the early years with an upward trend in later years (R^2^ = 0.02) (**Figure 4**). For ring width, *P. sylvestris* showed a continuous negative growth trend, whereas for *L. gmelinii* no clear pattern was observed, and it continued increasing and decreasing throughout (R^2^ = 0.19) (**Figure 4**). Lumen growth of both species at different sites across the years is also shown in (**Figure 5**).

**Figure 6:**
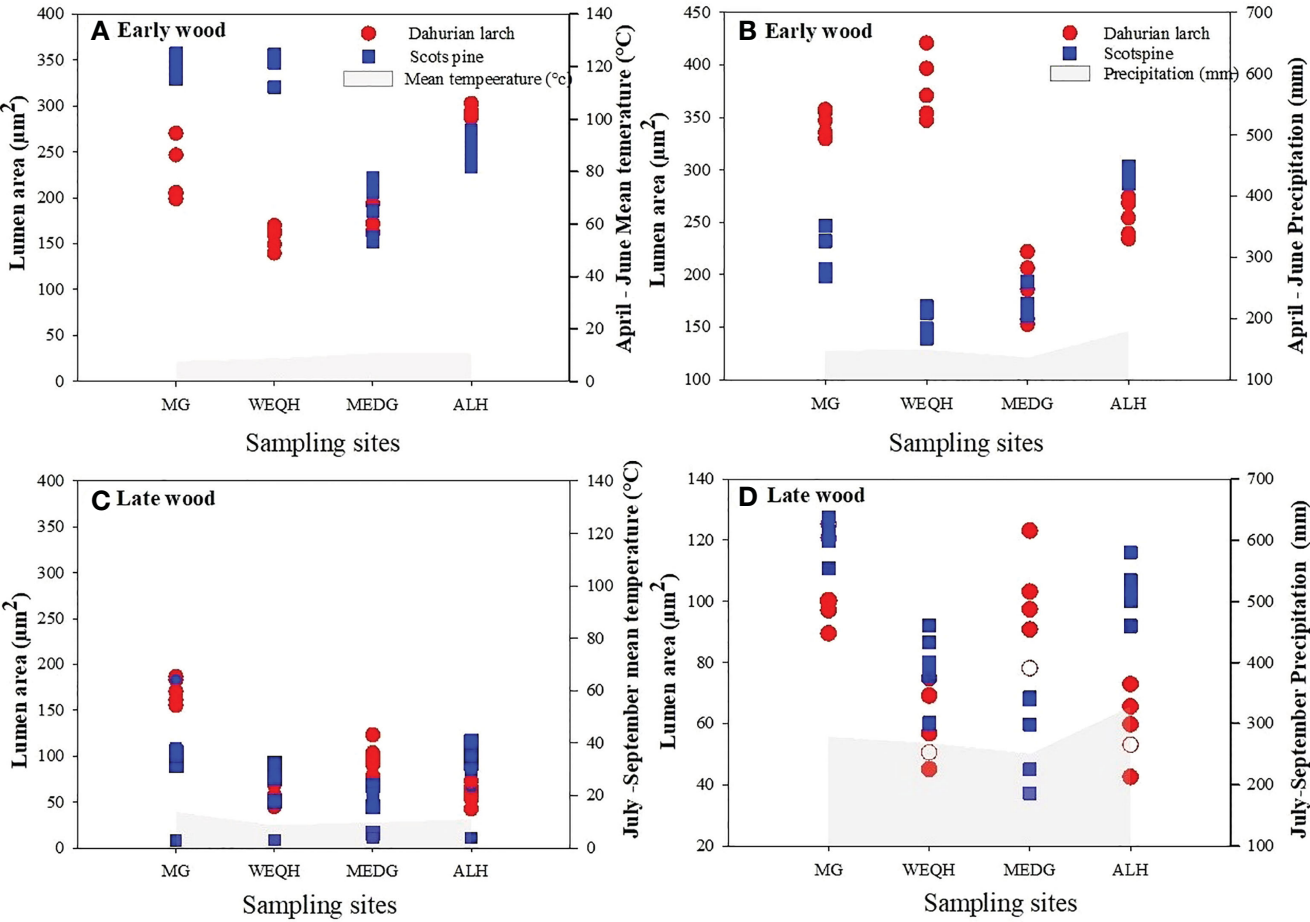
Relationship between mean lumen area and climatic variables (mean air temperature and total precipitation) during 2006-2012. April-June for earlywood, and July-September of latewood of *Pinus sylvestris* (Scots pine) and *Larix gmelinii* (Dahurian Larch) along four sites (MG, WEQH, MEDG, ALH).

Earlywood part of *L. gmelinii* showed a reduced lumen area with the increasing air temperature. In contrast, a positive correlation was found between earlywood and summer precipitation. Latewood showed a similar but weaker relationship with winter temperature for both species (**Figure 6**).

**Figure 7:**
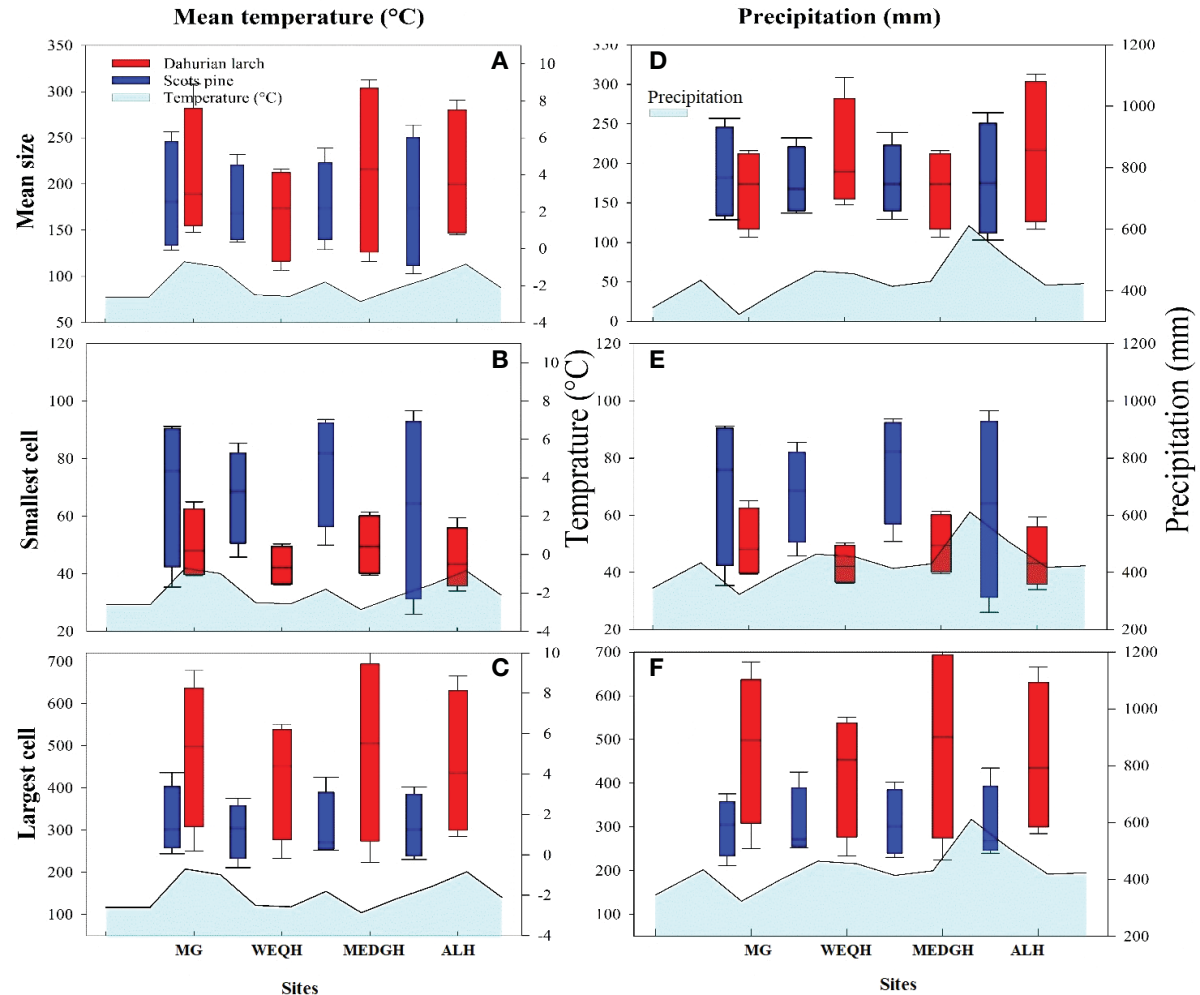
Box plots of mean temperature and precipitation with mean size, smallest cell, and largest cell of *Pinus sylvestris* (Scots pine) and *Larix gmelinii* (Dahurian Larch) species along four sites (MG, WEQH, MEDG, ALH).

**Figure 8:**
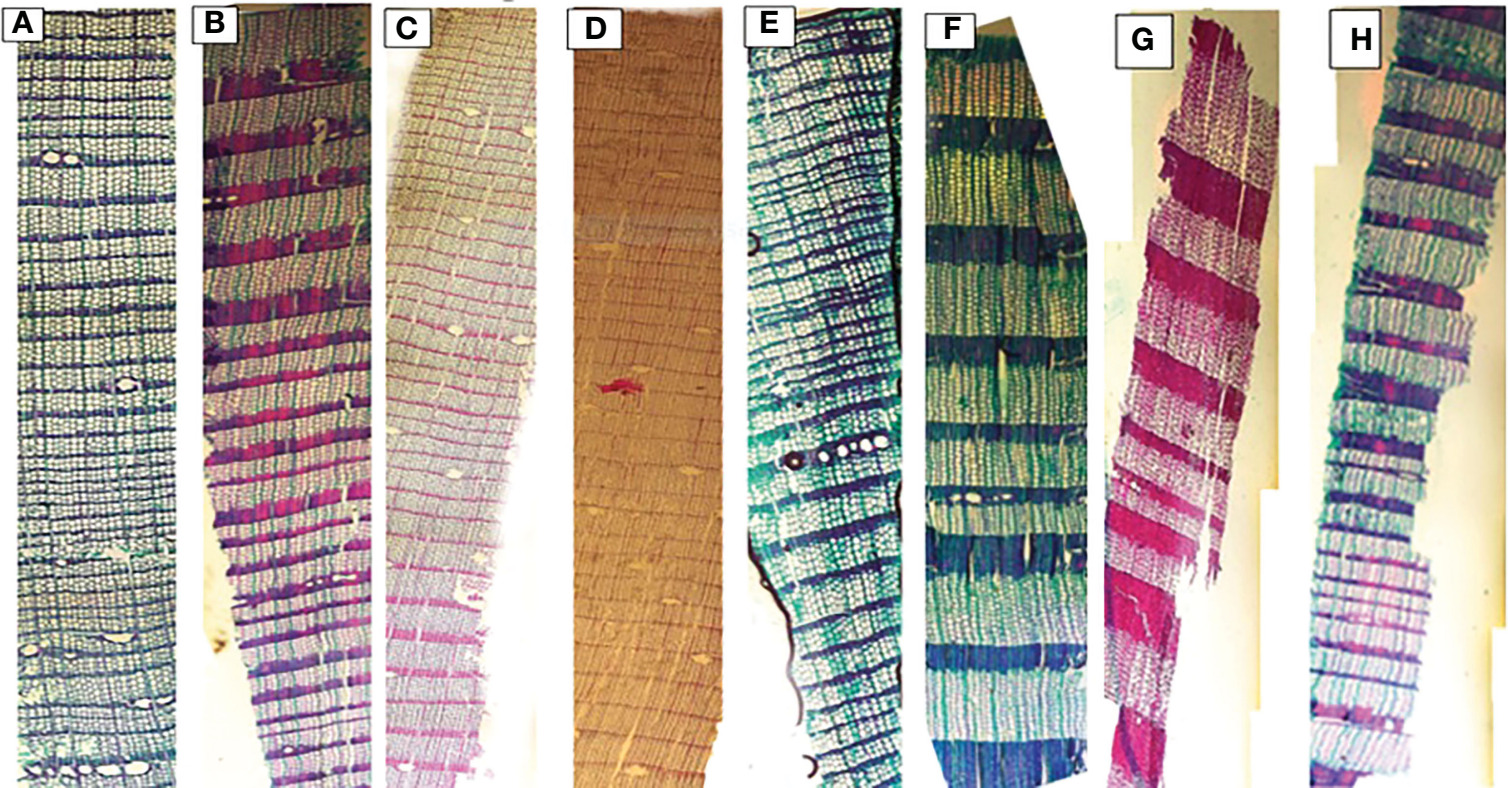
Xylem anatomical cross sections of *Pinus sylvestris* (Scots pine) and *Larix gmelinii* (Dahurian Larch) from four study sites (MG, WEQH, MEDG, ALH), respectively. Images shows the part of the whole tree of *P. sylvestris* **(A–D)** and *L. gmelinii* **(E–H)** at four sites in Daxing’an Mountains, respectively.

Significant differences were observed for mean, smallest and largest cell sizes among all sites for both species. Mean cell size growth for *L. gmelinii* represented more consistent growth (**Figures 7A, D**). However, in the smallest cell, there was quite the opposite response. There were much higher values for the smallest cell in the case of *P. sylvestris* (**Figures 7B, E**). In the case of *L. gmelinii*, larger cells have a similar growth response with temperature and precipitation at all four sites, while *P. sylvestris* had little growth of larger cells (**Figures 7C, F**). It was examined that *L. gmelinii* showed more growth response to temperature and *P. sylvestris* had a much higher response to precipitation. Images of microcores of the xylem tree ring in *L.gmelinii* and *P.sylvestrris* from the four study sites are shown in (**Figure 8**); differences in the images indicate the growth differences in both species at different sites.

## 4 Discussion

Regarding microclimate, temperature and precipitation are the two crucial ecological limiting factors that control the plant cell size by influencing growth parameters. Different studies reported that xylem growth and cambial activity highly respond to microclimatic factors (Deslauriers et al., 2008; Gruber et al., 2009; Fakhrutdinova et al., 2017). The Daxing’an mountains are one of the most apparent regions in China with a warming climate, especially in winter (Gao et al., 2019). Over the past few decades, increasing mean air temperature during May and June improved growing conditions. Even in the winter, the temperature is vital for the growth of trees in the following year.

In this study, the xylem anatomical growth of *L. gmelinii* and *P. sylvestris* presented different responses to microclimatic factors at different sites. Overall, mean temperature and precipitation significantly altered both species’ cell size dynamics. However, the effect of temperature on xylem anatomical growth was more prominent than precipitation. Zhang et al. (2018) have reported that variations in *L. gmelinii* and *P. sylvestris* were determined by moisture (negative) and temperature (positive) during the growing season. The temperature has the most prominent effect on the growth of *L. gmelinii* favours the results that the main limiting factor for the growth of *L. gmelinii* is the summer temperature (Bai et al., 2016; Yasmeen et al., 2019). Bai et al. (2016) also reported that summer temperatures significantly influence the survival and growth of conifers in northeastern China. In contrast, decreased precipitation was more responsive regarding root biomass and changing the soil microenvironment. Furthermore, the impact of water availability on the growth of *P. sylvestris* was more significant than that of *L. gmelinii.* Reich et al. (2018) showed that water deficiency in the soil might reduce or even reverse the climate warming benefits on net photosynthesis in cold environments, even with modest drought in the growing season.

The tracheid growth of *P. sylvestris* in our study area was more sensitive to precipitation. This effect was most significant at low-altitude sites. Eilmann et al. (2009) reported that more significant changes in the tracheid dimension had been observed in *P. sylvestris* due to increased drought severity in the Iberian Peninsula and some other conifers of the Alps range. Our results indicated that variations in wood density fractions are significant in determining xylem anatomical features and could explain the correlation between climate changes and tree growth (Carvalho et al., 2022). In this study, the high correlation between the earlywood and precipitation indicates a higher proportion of earlywood among the whole wood. Moreover, it was found that tree at higher elevations has double tree ring width than those found at lower elevations (Vaganov et al., 2006). Tree ring width is a sensitive part of the tree, reacting to any variations in growth conditions. Numerous anatomical studies have revealed that ring width changes significantly depend on the geographical region’s climate conditions (Fakhrutdinova et al., 2017).

This study observed a general reduction in the lumen area of cells in north-south directions in both conifer species from early to mid-summer. This is an adaptive strategy of trees to tolerate drought and reduce the risk of cavitation in the xylem. Small lumen areas can also result from the earlier onset of radial growth (Peltola et al., 2002; García-Cervigón et al.,2020). Moreover, a high growth rate and rapid cell division can also cause a narrow lumen area (Atwell et al., 2003). Usually, climatic variations cause an increase in the tracheid lumen area with a decrease in wood density at the end of the tree ring (Balzano et al., 2020).

In this study, the larger proportion consists of earlywood rather than latewood. The results of *P. sylvestris* growth in northern Finland revealed that 75% of the entire ring width part is earlywood (Seo et al., 2012). Our results are also in accordance with Park and Spiecker. (2005) and Olano et al. (2012) who stated that latewood generally acts as a buffer zone between tree rings in different years. It works like a “sponge” in which nutrients and minerals dissolved in water are reserved. The tree used these minerals in unfavorable weather conditions.

The time windows in which most variations best correlate with precipitation are usually smaller than temperature. April-June and July-September precipitation first increased and then decreased. Earlywood (average size of cell area or maximum length) showed more significant variations than latewood, where the precipitation was most significant. In the case of *P. sylvestris*, both temperature and precipitation caused an increase in early wood growth; however, in the case of latewood, there was a slight increase in temperature and a decline in precipitation within the months of July-September (Schmitt et al., 2004; Pritzkow et al., 2014). Considering the influence of the previous climate on the current growing season local climate conditions, temperature and precipitation showed the different growth patterns of *L. gmelinii* and *P. sylvestris* with the change in latitude (Yasmeen et al., 2019).

Our findings on climate parameters influence from previous July to current September during the period 2000-2016 are also consistent with the pines in Qinling Mountain (Liu et al., 2009), which showed a negative correlation from January and February. Particularly, climate-growth relationships performed separately on early and latewood. Because they were formed at different times, performed different functions, and helped to categorize the fundamental relationship of xylem plastic response and their possible role. Since 1970-1990, both *L. gmelinii* and *P. sylvestris* have decreased in growth, consistent with previous studies. Since 1990, there has been a recovery in the tree growth of *P. sylvestris*. However, the growth rate variation might depend on the ground vegetation thickness, which adjusts heat exchange between air and soil.

## 5 Conclusions

In this study, cell wall thickness and ring width showed a significantly varied response from the previous winter to the current growing season of two conifers species coexisting under boreal frost climate conditions. The positive effect of precipitation in summer, along with increasing latitude, is coordinated with the spatial distribution of precipitation. Drought stress was a secondary tree growth-limiting factor. Wood density parameters were strongly correlated with the temperature of the current year. Furthermore, the lumen size of *P. sylvestris* revealed a strong precipitation signal in latewood in July-September, which has not been so for observed in other species on that site. Xylem plasticity responses are due to water availability or deficiency conditions, which may be related to the accessibility (root system depth) of soil water reserves for different species, such as *L. gmelinii* has a shallow root system than *P. sylvestris*. Therefore, we need to explore further the plasticity of xylem anatomical parameters on more climates and species to determine the accurate range of xylem adjustment in changing environmental conditions. Our findings emphasize that the xylem anatomical-climate relationships of *P. sylvestris* and *L. gmelinii* shows a contrasting trend across different sites and climatic parameters, which means that these two coniferous species may exhibit an entirely opposite dendroanatomical response to climate change along the latitude gradient. There is one shortcoming in our study. Usually two cores are taken from one sample tree to avoid the sampling bias. However, in our study we only sampled one core from some sites. The reason is, in some special cases, to deal with topography, protect the integrity of the sample plots, to avoid tree damage, and economic considerations, it is still possible to collect one core per tree.

## Data availability statement

The original contributions presented in the study are included in the article/supplementary material. Further inquiries can be directed to the corresponding authors.

## Author contributions

TF, SY and QL conceived the idea and designed the experiment. SY, SA and MR conducted the study. SY, AS and MHR wrote the manuscript. MN and HL give suggestions to the manuscript. QL provide the funding. TF and QL supervise the experiment and reviewed the manuscript. All authors contributed to the article and approved the submitted version.
